# Supplementary material for: The Effect of Standardised Leaf Extracts of Gaultheria procumbens on Multiple Oxidants, Inflammation-Related Enzymes, and Pro-Oxidant and Pro-Inflammatory Functions of Human Neutrophils
Source: Molecules. 2022 May 23;27(10):3357. doi: 10.3390/molecules27103357 (PMC9144433; doi:10.3390/molecules27103357)
Supplement: Supplementary file 1 [file molecules-27-03357-s001.zip › molecules-1725632-supplementary.pdf]

molecules

Article

The Effect of Standardised Leaf Extracts of *Gaultheria procumbens* on Multiple Oxidants, Inflammation-Related Enzymes, and Pro-Oxidant and Pro-Inflammatory Functions of Human Neutrophils

Piotr Michel [1,*], Sebastian Granica [2], Karolina Rosińska [1], Małgorzata Glige [1], Jarosław Rojek [1], Łukasz Poraj [1], Monika Anna Olszewska [1]

[1] Department of Pharmacognosy, Faculty of Pharmacy, Medical University of Lodz, 1 Muszynskiego St., 90-151 Lodz, Poland; karolinaa.rosinskaa@gmail.com (K.R.); gosiaju11@gmail.com (M.G.); jaroslaw.rojek@o2.pl (J.R.); lukasz.poraj@gmail.com (Ł.P.); monika.olszewska@umed.lodz.pl (M.A.O.)

[2] Microbiota Lab, Centre for Preclinical Studies, Department of Pharmacognosy and Molecular Basis of Phytotherapy, Medical University of Warsaw, 1 Banacha St., 02-097 Warsaw, Poland; sgranica@wum.edu.pl (S.G.)

* Correspondence: piotr.michel@umed.lodz.pl (P.M.); Tel.: +48 426779169

Supplementary Materials:

Table S1. Phenolic analytes detected in *G. procumbens* leaf dry extracts by UHPLC-PDA-ESI-MS^3.

Table S2. Correlation (*r*) coefficients and probability (*p*) values of linear relationships between antioxidant and anti-inflammatory activity parameters and phenolic contents of *G. procumbens* leaf dry extracts.

Table S1. Phenolic analytes detected in *G. procumbens* leaf dry extracts by UHPLC-PDA-ESI-MS^3.

Peak	Analyte	t_R (min)	UV λ_{max} (nm)	[M–H]$^-$ *m/z*	MS^2 (% relative abundance)	MS^3 (% relative abundance)	Extract
1	protocatechuic acid hexoside	4.1	259, 293	315	225 (25); 153 (100); 107 (8)		All
2	protocatechuic acid [a]	4.6	259, 293	153			All
3	protocatechuic acid hexoside	5.6	259, 293	315	225 (1); 153 (100); 107 (2)		ME
4	unknown compound	5.8	310	331	313 (100); 211 (9); 167 (42); 125 (9)		All
5	3-*O*-caffeoylquinic acid (neochlorogenic acid) [a]	6.4	325	353	191 (100); 179 (50)		All
6	3-*O*-*p*-coumaroylquinic acid derivative	6.8	310	371	353 (6); 325 (72); 163 (100)		All
7	*p*-hydroxybenzoic acid [a]	7.6	254	137			EAE
8	procyanidin A-type dimer	9.4	280	575	499 (100); 490 (84); 451 (22); 407 (17); 289 (7)		All
9	3-*O*-*p*-coumaroylquinic acid hexoside	10.3	310	325	163 (100); 119 (9)		All
10	5-*O*-caffeoylquinic acid (chlorogenic acid) [a]	11.0	325	353	191 (100); 179 (5)		All
11	methyl salicylate 2-*O*-*β*-D-glucopyranosyl-(1→2)-[*O*-*β*-D-xylopyranosyl-(1→6)]-*O*-*β*-D-glucopyranoside [a, b]	12.6	285	653 *	607 (100); 575 (12)		ME, BE
12	4-*O*-caffeoylquinic acid (cryptochlorogenic acid) [a]	12.8	325	353	173 (100)		ME, BE
13	procyanidin B-type dimer	14.0	280	577	559 (17); 451 (41); **425 (100)**; 407 (44); 289 (27)	407 (100); 339 (2); 273 (7)	All
14	procyanidin B2 [a]	15.2	280	577	**425 (100)**; 407 (51); 289 (15)	407 (100); 273 (6)	ME, BE
15	procyanidin B-type trimer	15.5	280	865	847 (15); 739 (79); **713 (48)**; 695 (67); 577 (79); 451 (21); 287 (38)	695 (100); 575 (45); 561 (11); 425 (21); 405 (34); 287 (16)	All
16	gaultherin isomer [b]	15.7	285	491 *	445 (7); 413 (10); 293 (100); 149 (4)		All
17	unknown compound	16.2	280	417	373 (100); 331 (75)		ME, BE
18	(–)-epicatechin [a]	16.5	280	289	245 (100); 205 (19)		All
19	caffeoylquinic acid derivative	16.9	325	431	385 (100); 277 (7); 179 (22)		ME, BE
20	gaultherin [a]	17.1	285	491 *	445 (7); 413 (4); 293 (100); 149 (2)		All
21	unknown compound	17.7	280	363	345 (4); 183 (100); 179 (19); 143 (7); 121 (14)		All

Table S1. *Cont.*

Peak	Analyte	t_R (min)	UV λ_{max} (nm)	[M–H]⁻ *m/z*	MS² (% relative abundance)	MS³ (% relative abundance)	Extract
22	procyanidin A-type trimer	18.4	280	863	**711 (100)**; 573 (32); 559 (19); 451 (53); 411 (75); 289 (100)	693 (100); 559 (49); 541 (9); 407 (11)	ME, BE
23	procyanidin B-type dimer	19.3	280	577	559 (11); 451 (38); **425 (100)**; 407 (46); 289 (36)	407 (100); 381 (13); 299 (18); 273 (4)	All
24	procyanidin A-type trimer (cinnamtannin B-1) [a, b]	20.0	280	863	**711 (100)**; 693 (11); 573 (23); 559 (15); 451 (16); 411 (13); 289 (5)	693 (79); 559 (100); 541 (33); 463 (14); 407 (26)	All
25	procyanidin B-type trimer (procyanidin C1) [a, b]	21.2	280	865	847 (36); 739 (71); **713 (45)**; 695 (100); 577 (95); 451 (19); 287 (20)	693 (100); 561 (64); 411 (19); 243 (4)	ME, BE
26	procyanidin A-type trimer	21.6	280	863	**711 (100)**; 573 (41); 559 (10); 451 (53); 411 (78); 289 (25)	693 (100); 559 (79); 541 (28); 463 (19); 407 (21)	ME, BE
27	caffeoylquinic acid derivative	22.2	325	391	225 (17); 179 (100); 161 (4); 143 (9); 119 (7); 113 (6)		All
28	procyanidin A-type dimer	22.4	280	575	499 (100); 491 (4); 451 (8); 423 (16); 289 (22)		All
29	procyanidin A-type dimer	23.4	280	575	499 (100); 491 (67); 451 (11); 423 (24); 289 (73)		ME, BE
30	procyanidin A-type dimer	24.7	280	575	499 (100); 491 (21); 451 (13); 423 (18); 289 (37)		All
31	quercetin 3-*O*-*β*-D-xylopyranosyl-(1→2)-*β*-D-glucuronopyranoside (wintergreenoside A) [a, b]	25.5	257, 356	609	477 (2); **301 (100)**	273 (47); 255 (17); 179 (100); 151 (79)	ME, BE
32	procyanidin A-type dimer	26.4	280	575	499 (23); 491 (16); 451 (6); 411 (57); 289 (100)		ME, BE
33	procyanidin A-type trimer	26.8	280	863	**711 (100)**; 573 (58); 559 (9); 451 (58); 411 (87); 289 (29)	693 (100); 559 (11); 541 (15); 463 (42); 407 (28)	All
34	3-*O*-*p*-coumaroylquinic acid derivative	27.2	310	487	441 (100); 307 (73); 163 (7)		ME, EAE
35	quercetin 3-*O*-*β*-D-galactopyranoside (hyperoside) [a]	27.6	254, 353	463	**301 (100)**	273 (49); 255 (19); 179 (95); 151 (75)	All
36	quercetin 3-*O*-*β*-D-glucopyranoside (isoquercitrin) [a]	28.5	256, 353	463	**301 (100)**	273 (53); 255 (25); 179 (100); 151 (71)	All
37	quercetin 3-*O*-*β*-D-glucuronopyranoside (miquelianin) [a]	29.1	256, 356	477	**301 (100)**	273 (17); 257 (16); 179 (100); 151 (61)	All

Table S1. *Cont.*

Peak	Analyte	t_R (min)	UV λ_{max} (nm)	[M–H]⁻ *m*/*z*	MS² (% relative abundance)	MS³ (% relative abundance)	Extract
38	kaempferol 3-*O*-*β*-D-xylopyranosyl-(1→2)-*β*-D-glucuronopyranoside (wintergreenoside B) [a, b]	29.4	275, 345	593	**285 (100)**	267 (31); 257 (100); 241 (19); 229 (35); 151 (19)	ME, BE
39	quercetin 3-*O*-*α*-L-arabinopyranoside (guaijaverin) [a]	30.8	258, 356	433	**301 (100)**	273 (45); 255 (17); 179 (100); 151 (73)	All
40	kaempferol 3-*O*-*β*-D-glucuronopyranoside [a]	33.3	265, 345	461	**285 (100)**	267 (39); 257 (100); 241 (40); 229 (31); 151 (9)	ME, BE
41	quercetin [a]	43.7	255, 364	301	273 (41); 255 (18); 179 (100); 151 (74)		All

[a] confirmed by comparison with authentic standard; [b] detected for the first time in *G. procumbens* leaf; t*R*, retention times; UV λ_{max}, absorbance maxima in PDA spectra; $[M-H]^-$, pseudomolecular ions in MS spectra recorded in a negative ion mode; in bold – ions subjected to MS^3 fragmentation; * $[M+HCOO]^-$. The nomenclature of caffeoylquinic acids isomers is according to IUPAC.

Table S2. Correlation (*r*) coefficients and probability (*p*) values of linear relationships between antioxidant and anti-inflammatory activity parameters and phenolic contents of *G. procumbens* leaf dry extracts.

***r* (*p*) for:**	**Antioxidant activity**						**Anti-inflammatory activity**		
	**DPPH**	**FRAP**	**TBARS**	**$O_2^{\bullet-}$**	**$^{\bullet}OH$**	**H_2O_2**	**HYAL**	**LOX**	**COX-2**
**TPC**	–0.6961 (0.510)	0.9841 (0.114)	–0.7996 (0.410)	–0.7459 (0.464)	–0.7302 (0.479)	–0.5171 (0.654)	–0.3675 (0.760)	–0.3351 (0.782)	–0.8080 (0.401)
**TPH**	0.9447 (0.213)	–0.5767 (0.609)	0.8820 (0.312)	–0.2889 (0.813)	0.9276 (0.244)	0.9942 (0.068)	0.9982 (0.038) *	0.9956 (0.060)	0.8753 (0.321)
**TPA**	–0.8892 (0.302)	0.9892 (0.094)	–0.9497 (0.203)	–0.4935 (0.671)	–0.9104 (0.271)	–0.7638 (0.447)	–0.6458 (0.553)	–0.6190 (0.575)	–0.9540 (0.194)
**TLPA**	–0.9841 (0.114)	0.9032 (0.282)	–0.9998 (0.014) *	–0.2178 (0.860)	–0.9916 (0.083)	–0.9191 (0.258)	–0.8407 (0.364)	–0.8215 (0.386)	–1.0000 (0.005) **
**TPHA**	0.8973 (0.291)	–0.4719 (0.687)	0.8174 (0.391)	–0.4043 (0.735)	0.8747 (0.322)	0.9735 (0.147)	0.9980 (0.040) *	0.9996 (0.018) *	0.8092 (0.400)
**TSAL**	0.9985 (0.035) *	–0.7795 (0.431)	0.9777 (0.135)	–0.0138 (0.991)	0.9947 (0.066)	0.9853 (0.109)	0.9431 (0.216)	0.9311 (0.238)	0.9747 (0.144)
**TFL**	–0.7831 (0.427)	0.9988 (0.031)	–0.8705 (0.328)	–0.6535 (0.547)	–0.8124 (0.396)	–0.6234 (0.571)	–0.4847 (0.678)	–0.4541 (0.700)	–0.8774 (0.319)

TPC: total phenolic content (Folin-Ciocalteau assay) in gallic acid equivalents, TPH: total phenolic content (HPLC), TSAL: total content of salicylates (HPLC), TPA: total content of proanthocyanidins (*n*-butanol/HCl assay) in cyanidin chloride equivalents, TLPA: total content of proanthocyanidins (HPLC), TPHA: total content of phenolic acids (HPLC), TFL: total content of flavonoids (HPLC). Asterisks mean significance of the estimated linear relationship (*$p < 0.05$, ** $p < 0.01$) for three extracts ($n = 3$).
